# Opium Use and Cancer Risk: A Comprehensive Systematic Review and Meta-Analysis of Observational Studies

**DOI:** 10.1155/2022/5397449

**Published:** 2022-02-18

**Authors:** Masoume Mansouri, Sina Naghshi, Mahbobeh Parsaeian, Sadaf G Sepanlou, Hossein Poustchi, Zahra Momayez Sanat, Omid Sadeghi, Akram Pourshams

**Affiliations:** ^1^Digestive Disease Research Center, Digestive Disease Research Institute, Tehran University of Medical Sciences, Shariati Hospital, Tehran, Iran; ^2^Students' Scientific Research Center, Tehran University of Medical Sciences, Tehran, Iran; ^3^Department of Clinical Nutrition, School of Nutritional Sciences and Dietetics, Tehran University of Medical Sciences, Tehran, Iran; ^4^Department of Biostatistics, Tehran University of Medical Sciences (TUMS), Tehran, Iran; ^5^Food Security Research Center, Department of Community Nutrition, School of Nutrition and Food Science, Isfahan University of Medical Sciences, Isfahan, Iran

## Abstract

**Background:**

Epidemiological studies have reported inconsistent associations between opium use and cancer risk. We therefore conducted a systematic review and meta-analysis to investigate the relationship between opium use and cancer risk.

**Methods:**

We searched PubMed, Scopus, ISI Web of Knowledge, and Google Scholar until February 2021 and references of retrieved relevant articles for observational studies that reported the risk of cancer in relation to opium use. Random-effects models were used to calculate pooled effect sizes (ESs) as well as 95% confidence intervals (CIs) for the association between opium use and cancer risk by considering opium doses and types, duration of consumption, and routes of opium use.

**Results:**

In total, 21 observational articles, with a total sample size of 64,412 individuals and 6,658 cases of cancer, were included in this systematic review and meta-analysis. Ever opium users, compared with never opium users, had 3.53 times greater risk of overall cancer (pooled ES: 3.53, 95% CI: 2.60–4.79, *P* ≤ 0.01). This positive association was also seen for some individual types of cancers except for esophageal and colon cancers. Also, we found that higher opium doses and higher duration of consumption were associated with an increased risk of overall and individual types of cancer. However, the associations between opium doses and the risk of head and neck and larynx cancers were not significant. In terms of the routes of opium use, both opium ingestion and smoking were positively associated with the risk of cancer. Regarding opium types, we found that using teriak, but not shireh, could increase the risk of cancer.

**Conclusions:**

Our findings showed that opium use, particularly in the form of teriak, is a risk factor for cancer.

## 1. Introduction

Opium, the raw extract of opium poppy, is an addictive substance that has been used for recreational or medical purposes [1]. It has been estimated that 16.5 million individuals around the world are addicted to different types of opiates; of them, 4 million people use raw opium [2, 3]. These people are mostly from Asia, while the prevalence of addiction to opium is low among western countries because of the criminalization of opium use and availability of other psychoactive drugs [2]. Opium may be prescribed for patients due to its analgesic, hypnotic, antitussive, and antidiarrheal effects [1, 4]. However, the health hazards of opium misuse have raised concerns about the long-term effects of opium use [5, 6].

An older generation of physicians and researchers believed that a low-dose consumption of opium for a long time can increase longevity by reducing the risk of chronic diseases such as diabetes mellitus, cardiovascular diseases, and cancer [2, 7]. This belief might be explained by the analgesic effect of opium. Contrary to this belief, recent publications have shown a positive relationship between opium use and some of the mentioned chronic diseases [5, 6]. Among these diseases, cancer has received great attention [8–28]. It has been proposed that opium consumption produces carcinogenic compounds such as heterocyclic and polycyclic aromatic hydrocarbons, primary aromatic amines, and N-nitrosamines [29, 30]. Despite the proposed mechanisms, findings from prospective and retrospective studies are conflicting. Some studies have shown a positive association between opium use and some cancers such as bladder and lung cancers [18, 26, 27] and some others reported that the link between opium use and cancer risk is due to cigarette that is usually smoked along with opium [16, 23]. Nevertheless, there are reports on the null association between opium consumption and cancer risk [8, 10]. In addition, it is not clear whether opium types and doses, as well as the duration and routes of using opium (smoking or ingestion), are involved in the carcinogenic effects of opiates or not.

Overall, there is a need for a comprehensive meta-analysis to reveal the above-mentioned uncertainties. A 2017 meta-analysis summarized available findings on the link between opium use and bladder cancer [31]; however, it was not comprehensive and did not take into consideration all types of cancers. Therefore, this comprehensive systematic review and meta-analysis of observational studies was conducted to summarize current evidence on the association between opium use and cancer risk by taking into account types, dosage, routes, and duration of opium use.

## 2. Methods

This systematic review and meta-analysis was conducted and reported according to the Preferred Reporting Items for Systematic Review and Meta-Analysis (PRISMA) guidelines [32].

### 2.1. Search Strategy

We performed a systematic search in the online databases of PubMed, Scopus, ISI Web of Science, and Google Scholar to find eligible papers which were published up to January 2021 and investigated the association between opium use and cancer risk. In the search strategy, appropriate keywords including MeSH (medical subject heading terms) and non-MeSH terms were used (Supplemental Table 1). We took into consideration no restrictions in terms of publication time or the language of papers. Furthermore, the reference lists of the included papers and recent reviews were reviewed to detect articles not found in the literature search.

### 2.2. Inclusion Criteria

The studies with the following criteria were included: (1) studies with prospective or retrospective (i.e., case-control) design; (2) those that were conducted on adults (≥18 years); (3) studies that evaluated opium use or different aspects of using opium (i.e., types, doses, routes, and duration of opium use) in relation to cancer risk, whether overall cancer or specific cancers; (4) articles that reported relative risk (RR), hazard ratio (HR), or odds ratio (OR) with 95% confidence intervals (CI) for the relation between opium use and cancer risk. If results from one study were published in >1 paper, we selected the most recent one; otherwise, the one with the greatest number of cases or with higher quality was included.

### 2.3. Exclusion Criteria

In the current meta-analysis, we did not include letters, comments, retracted articles, reviews, and ecological studies. Also, studies with insufficient data, those that were conducted on children and adolescences, studies with a cross-sectional design, and those with duplicate results were excluded.

### 2.4. Data Extraction

Required data for the systematic review and meta-analysis were extracted from each paper by two independent investigators. Any reported effect sizes (ESs) including ORs, RRs, or HRs along with 95% CIs for the relation between opium use and cancer risk were extracted to be used in the meta-analysis. For articles with several ESs for one association, the one that was adjusted for the most confounding variables was extracted. Moreover, additional information on the first author name, publication year, study design, sample size, number of cases, demographic characteristics of participants (age range or mean age, gender, and health condition), study location, duration of follow-up (for prospective studies), methods used to assess opium consumption or cancer, and confounding variables adjusted in the statistical analysis was extracted from each included article.

### 2.5. Risk of Bias Assessment

The quality of included studies was determined using the Newcastle-Ottawa Scale (NOS), designed for nonrandomized studies [33]. Based on this scale, an article can get a maximum of 9 scores given the following parameters: 4 scores for the selection of participants, 2 scores for comparability, and 3 scores for the assessment of outcomes. When a study received more than median scores, it was deemed to be of relatively high quality (or low risk of bias); otherwise, it was considered to be of low-quality (or high risk of bias) article.

### 2.6. Statistical Methods

We included the ORs, RRs, and HRs (and 95% CIs) reported for the association between opium use and cancer risk into the meta-analysis. These ESs were reported for cancer risk in relation to ever versus never use of opium, doses and duration of consumption (the highest versus lowest doses and duration), routes of opium (smoking or ingestion versus never use of opium), and types of opiates [raw opium (teriak) or refined opium (shireh) versus never use of opium]. To perform meta-analysis, we first calculated the natural log form (and its standard error) of the ESs (ORs, RRs, and HRs), and then we combined them using a random-effects model that takes between-study heterogeneity into account [34]. If a paper presented the ESs based on gender or any other variables, we first combined them using a fixed-effects model, and then we included the pooled ES in the meta-analysis. By the random-effects model, we calculated both Q-statistic and *I*^2^ values as the indicators of heterogeneity. *I*^2^ values >50% were considered to indicate significant between-study heterogeneity [35]. In the case of significant heterogeneity, we performed subgroup analyses based on predefined criteria including study design (prospective versus case-control studies), methods used for cancer ascertainment (histological or pathological methods versus medical records), quality of studies (high quality versus low quality), and statistical adjustments for tobacco use and dietary factors (adjusted versus not adjusted). Publication bias was determined using Egger's linear regression test [36]. In the case of substantial publication bias, the trim-and-fill method was used to detect the effect of probable missing studies on the overall ES [37]. To assess if the overall ES depended on one study, the sensitivity analysis was conducted using a random-effects model in which each study was excluded to determine the influence of that study on the overall estimate. Statistical analyses were conducted using STATA version 14.0. *P* < 0.05 was considered as statistically significant for all tests.

## 3. Results

### 3.1. Literature Search

**Figure 1:**
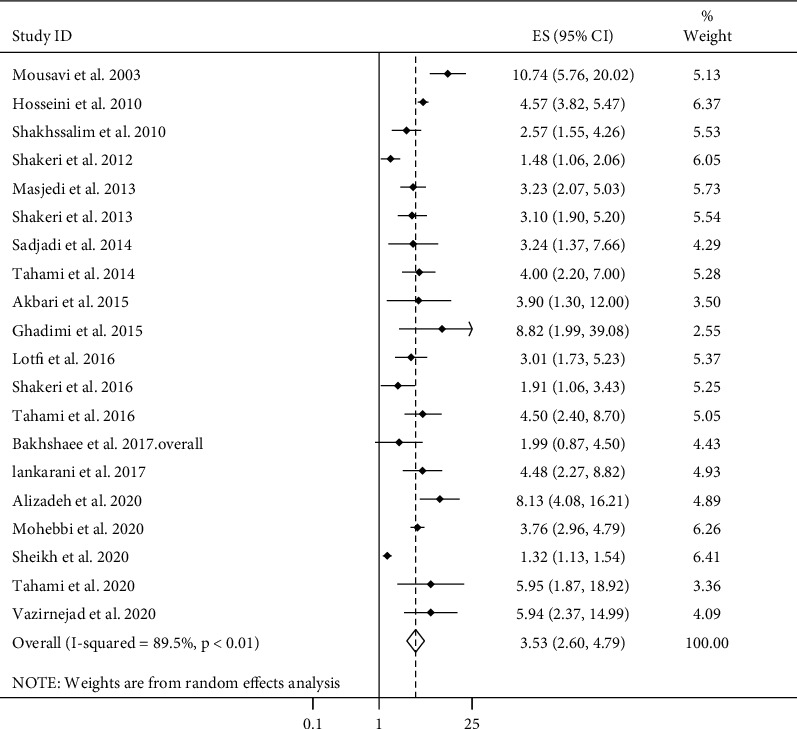
Forest plot for the association between opium use and cancer risk in adults aged ≥18 years by comparing ever with never users of opium. ES: effect size.

By searching relevant keywords in the online databases, we found 600 articles. We excluded duplicate papers and studies that did not meet the inclusion criteria and, finally, 30 full articles of potentially relevant studies remained for further assessment (Figure 1). Of the 30 papers, six reported no effect sizes for the relation between opium use and cancer risk and, therefore, were excluded [38–43]. Two articles were published on a case-control dataset [15, 44] and three other articles were published on the dataset of Golestan Cohort Study [27, 45, 46]. Since these articles assessed similar exposure and outcome variables, we included only the one with higher quality or with the most number of cases for each dataset [15, 27] and excluded the duplicate papers [44–46]. After the exclusions, 21 papers with full data were included in the present systematic review and meta-analysis [8–28].

### 3.2. Characteristics of Included Studies

Characteristics of studies included in the current systematic review and meta-analysis are shown in Supplemental Table 2. All papers were published between 2003 and 2020. Two articles had a prospective design [22, 27] and the rest of the papers were of case-control design. The number of participants in these studies ranged from 181 to 50,045 subjects aged 18 years and older. In total, 64,412 subjects with 6,658 cases of cancer were included in the 21 papers we assessed. In all studies, both males and females were recruited. In addition, all studies were conducted in the Middle East, Iran. Among the 21 articles, different types of cancers including gastrointestinal (GI) [10, 13, 16, 18, 20–25, 27, 28], bladder [8, 11, 12, 14, 26, 27], lung [15, 19, 27], head and neck [9, 16, 27], ovarian [27], prostate [27], cervical [27], and breast [27] cancers, as well as leukemia and lymphoma [27], were studied. In addition, among GI cancers, the risk of gastric [18, 22, 25, 27], esophageal [10, 21, 24, 27], oral and pharynx [16, 27], pancreatic [23, 27], liver [27], and colorectal cancers [13, 20, 27] was assessed. Cancer ascertainment was done using histological or pathological methods in fifteen articles [10–17, 21–25, 27, 28], while six articles used the information from medical records for this purpose [8, 9, 18–20, 26].

In terms of exposure, opium use was evaluated using interview-based questionnaires in all studies. All articles except one [21] presented risk estimates for ever versus never consumers of opium, 11 papers considered duration of opium use as an exposure variable [8, 9, 13, 15, 16, 18–20, 23, 24, 27], and some publications assessed doses [8, 9, 13, 15, 16, 18–21, 23] and types [16, 21, 27] of opiates in relation to cancer risk. Furthermore, the routes of opium use were investigated with cancer risk in five papers [12, 15, 16, 21, 27]. In the most included publications, some important confounders including age (*n* = 20), tobacco use (*n* = 16), dietary factors (*n* = 9), and alcohol consumption (*n* = 6) were adjusted in the analysis. Based on the NOS tool and by considering the median score of 7 among the included studies, 17 papers were of high-quality studies and the remaining 5 articles were considered as low-quality publications (Supplemental Table 3).

### 3.3. Findings from the Systematic Review

Of 20 articles that compared ever with never consumers of opium, 17 publications revealed a positive association between opium use and cancer risk [8, 9, 11–15, 17–20, 22–26, 28], whereas 3 papers found evidence of a nonsignificant association [10, 16, 27]. In terms of opium doses, 7 papers revealed that high doses of opiates were associated with an increased risk of cancer [9, 13, 15, 18–21] and other articles showed a null association between opium doses and cancer risk [8, 16, 23]. Regarding the duration of opium use, seven papers indicated a positive association with cancer risk [8, 9, 13, 15, 18–20] and three articles did not find any significant association [16, 23, 24]. Also, in a cohort study [27], the authors concluded that the association between duration of opium use and cancer risk depended on cancer type: a positive association with non-GI cancers and a null association with overall risk of GI cancer. Two papers showed that the association between opium use and cancer did not depend on the routes of consumption (smoking versus ingestion) [12, 21]; however, three articles presented different results for opium smoking and ingestion [15, 16, 27]. Based on their findings, opium smoking was associated with an increased risk of lung, pharynx, gastric, esophageal, and laryngeal cancers, whereas opium ingestion was associated with a greater risk of liver and oral cancers. Three papers assessed the types of opiates (teriak and shireh) in relation to cancer risk; of them, two revealed different results for each type of opiates [16, 27] and one reported the same result for both types [21].

### 3.4. Findings from the Meta-Analysis

All studies included in the systematic review contained complete data needed for the meta-analysis. Meta-analysis findings are reported separately for each type of exposure.

### 3.5. Ever versus Never Use of Opium and Risk of Cancer

Combining twenty ESs from 20 articles [8–20, 22–28] regarding overall cancer risk, we found a significant positive association between ever use of opium and overall cancer risk (pooled ES: 3.53, 95% CI: 2.60–4.79, *P* ≤ 0.01) (Figure 1). However, heterogeneity between studies was significant (*I*^2^ = 89.5%, *P* ≤ 0.01). We performed subgroup analyses to detect possible sources of heterogeneity (Table 1). Subgroup analyses based on methods used to assess cancer and statistical adjustments for dietary factors and tobacco use could explain the between-study heterogeneity. In all subgroups of studies, except prospective cohort studies, we found a significant positive relationship between ever opium use and overall cancer risk. Regarding different types of cancers, ever opium use was associated with an increased risk of GI (pooled ES: 2.49, 95% CI: 1.81–3.43, *P* ≤ 0.01), bladder (pooled ES: 3.85, 95% CI: 2.96–5.00, *P* ≤ 0.01), head and neck (pooled ES: 4.35, 95% CI: 2.61–7.26, *P* ≤ 0.01), lung (pooled ES: 5.00, 95% CI: 2.70–9.28, *P* ≤ 0.01), and larynx (pooled ES: 8.85, 95% CI: 6.16–12.74, *P* ≤ 0.01) cancers. For GI cancers, a similar finding was observed for oral, gastric, pancreas, and colorectal cancers, but the associations of ever opium use with esophageal and colon cancers were not significant. Data for other types of cancers were insufficient for performing the meta-analysis.

### 3.6. Opium Doses and Cancer Risk

**Figure 2:**
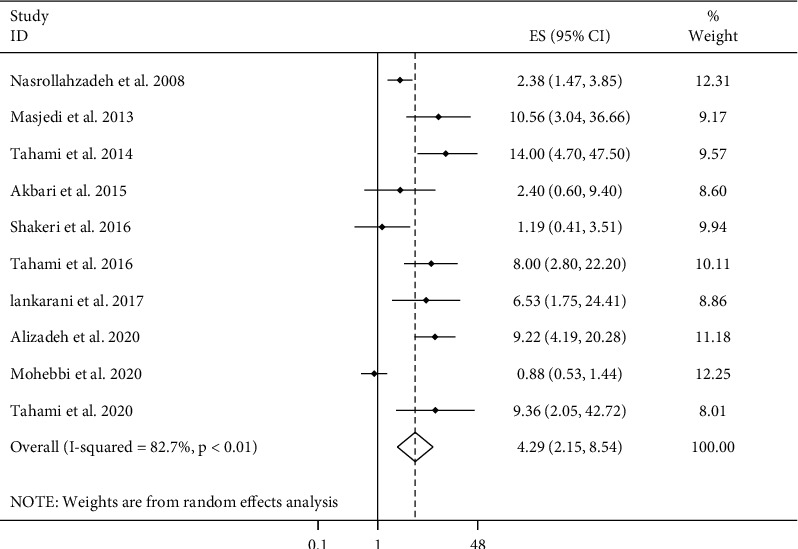
Forest plot for the association between opium dose and cancer risk in adults aged ≥18 years by comparing the highest with lowest consumption of opium. ES: effect size.

There were ten papers with ten ESs in this regard [8, 9, 13, 15, 16, 18–21, 23]. Combining these ESs, comparing the highest with lowest doses of opium use, a significant positive association was found with overall cancer risk (pooled ES: 4.29, 95% CI: 2.15–8.54, *P* ≤ 0.01) with a significant between-study heterogeneity (*I*^2^ = 82.7%, *P* ≤ 0.01) (Figure 2). Findings from the subgroup analyses (Table 1) showed that this heterogeneity might be due to differences of included studies in terms of methods used for cancer ascertainment and controlling for dietary factors in their analysis. In addition, the positive association between opium doses and overall cancer risk was significant in all subgroups of studies except those studies that did not control for dietary factors in their analysis. When the analyses were confined to individual types of cancers, we found that higher doses of opium use were associated with greater risk of GI (pooled ES: 2.82, 95% CI: 1.31–6.05, *P* ≤ 0.01), lung, colorectal, and colon cancers; however, this relationship became nonsignificant for head and neck and larynx cancers. It should be noted that, except for GI cancers that had six studies, other mentioned cancers were analyzed with only two studies.

### 3.7. Duration of Opium Use and Cancer Risk

**Figure 3:**
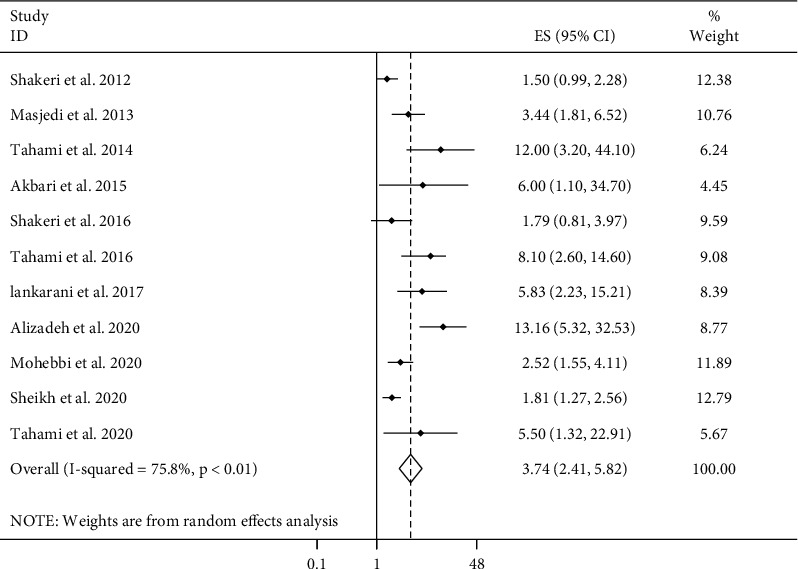
Forest plot for the association between duration of opium use and cancer risk in adults aged ≥18 years by comparing the longest with shortest duration of opium use. ES: effect size.

Considering 11 ESs from 11 papers [8, 9, 13, 15, 16, 18–20, 23, 24, 27], comparing the highest versus the lowest duration of opium use, we found that higher duration of opium use was associated with 3.74 times greater risk of overall cancer (95% CI: 2.41–5.82, *P* ≤ 0.01) (Figure 3). There was evidence of a significant between-study heterogeneity in this association (*I*^2^ = 75.8%, *P* ≤ 0.01). In the subgroup analyses, categorizing studies based on methods used for cancer assessment and statistical adjustments for dietary factors could decrease the observed heterogeneity (Table 1). In all subgroups of studies, the positive association between the duration of opium use and overall risk of cancer remained significant. In terms of cancer types, when combining seven studies for GI cancers [13, 16, 18, 20, 23, 24, 27] and three studies for lung cancer [15, 19, 27], a significant positive association was seen with the duration of opium use (GI cancers, pooled ES: 2.86, 95% CI: 1.69–4.83, *P* ≤ 0.01; lung cancer, pooled ES: 3.95, 95% CI: 2.37–6.60, *P* ≤ 0.01). Also, meta-analysis of two studies for each of head and neck [9, 16], larynx [9, 16], colorectal [13, 20], and colon [13, 20] cancers revealed such a positive relationship.

### 3.8. Routes of Opium Use and Cancer Risk

**Figure 4:**
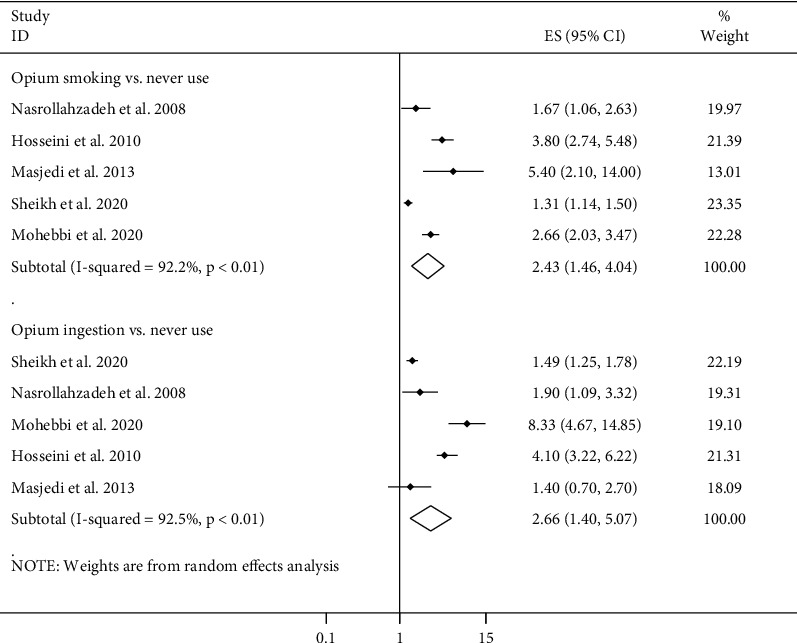
Forest plot for the association between routes of opium use and cancer risk in adults aged ≥18 years by comparing opium smoking or ingestion with never use of opium. ES: effect size.

In total, five articles assessed the routes of opium use (smoking and ingestion) in relation to overall cancer risk [12, 15, 16, 21, 27]. Combining five ESs from these studies, comparing opium ingestion or smoking with never use of opium, indicated that both routes of opium use were associated with an increased risk of overall cancer (opium smoking, pooled ES: 2.43, 95% CI: 1.46–4.04, *P* ≤ 0.01; opium ingestion, pooled ES: 2.66, 95% CI: 1.40–5.07, *P* ≤ 0.01) (Figure 4). Although between-study heterogeneity was significant in these associations (*I*^2^ > 90%, *P* ≤ 0.01), a limited number of studies did not allow us to find sources of heterogeneity using subgroup analysis. For different types of cancers, combining three studies for GI cancers [16, 21, 27] and two studies for bladder [12, 27], head and neck [16, 27], lung [15, 27], larynx [16, 27], and oral [16, 27] cancers showed a pooled ES of 1.47 (95% CI: 1.07–2.02, *P* = 0.01), 3.55 (95% CI: 2.59–4.86, *P* ≤ 0.01), 1.45 (95% CI: 0.40–5.35, *P* = 0.57), 3.00 (95% CI: 1.12–7.98, *P* = 0.03), 3.66 (95% CI: 2.24–5.97, *P* ≤ 0.01), and 1.71 (95% CI: 0.88–3.32, *P* = 0.11) for these cancers, respectively, when comparing opium smokers versus never opium users (Table 1). The same studies as mentioned for opium smoking presented ESs for comparing opium ingestion with never use of opium. Combining these ESs indicated that opium ingestion was associated with an increased risk of GI (pooled ES: 1.82, 95% CI: 1.15–2.89, *P* = 0.01), bladder (pooled ES: 4.02, 95% CI: 2.96–5.47, *P* ≤ 0.01), head and neck (pooled ES: 4.29, 95% CI: 1.11–16.62, *P* = 0.03), lung (pooled ES: 2.00, 95% CI: 1.05–3.83, *P* = 0.04), and larynx (pooled ES: 6.82, 95% CI: 1.05–44.17, *P* = 0.04) cancers. Such finding was not seen for oral cancer (Table 1).

### 3.9. Opium Types and Cancer Risk

**Figure 5:**
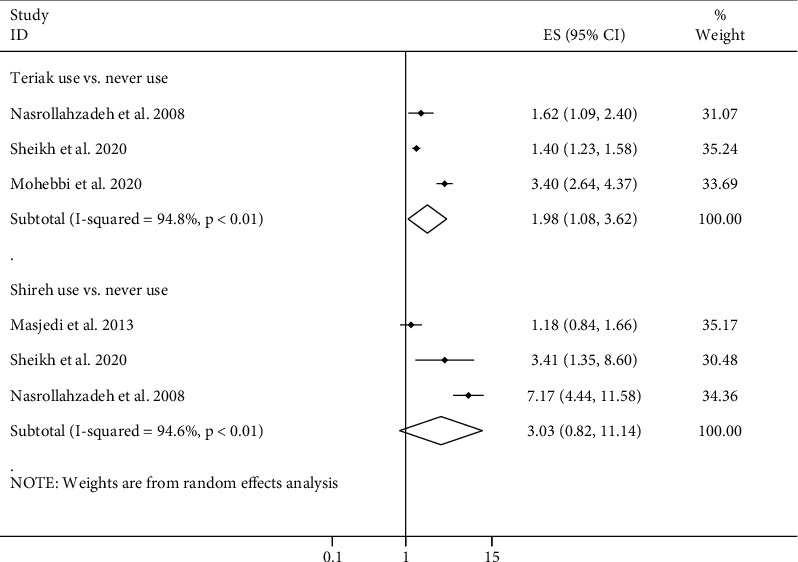
Forest plot for the association between routes of opium use and cancer risk in adults aged ≥18 years by comparing teriak and shireh use with never use of opium. ES: effect size.

In the present meta-analysis, we assessed the use of teriak as a raw opium and shireh as a refined opium in relation to cancer risk. Data on the other types of opiates were insufficient for doing a meta-analysis. In total, three papers provided data on the link between opium type and overall cancer risk [16, 21, 27]. Combining ESs from these papers revealed that using teriak, but not shireh, was associated with an increased risk of overall cancer compared with never use of opium (pooled ES: 1.98, 95% CI: 1.08–3.62, *P* = 0.03) (Figure 5). There was evidence of a significant heterogeneity between studies for both associations (*I*^2^ > 90%, *P* ≤ 0.01). Unfortunately, due to the limited number of published papers, subgroup analyses in these associations were not possible. Regarding specific types of cancer, we had three papers for GI cancers [16, 21, 27] and two articles for larynx cancer [16, 27] in relation to teriak or shireh use. In addition, two papers had ESs of association between teriak use and the risk of head and neck cancers [16, 27]. Combining ESs from these papers, we found that teriak users, compared with never opium users, had an increased risk of GI (pooled ES: 1.98, 95% CI: 1.08–3.62, *P* = 0.03) and larynx (pooled ES: 3.97, 95% CI: 1.69–9.36, *P* ≤ 0.01) cancers and also shireh users had an increased risk of larynx cancer compared with never opium users (pooled ES: 7.54, 95% CI: 2.13–26.63, *P* ≤ 0.01) (Table 1). No significant association was seen between shireh use and the risk of cancer (Figure 5).

### 3.10. Publication Bias and Sensitivity Analysis

For all associations assessed in the current meta-analysis, no publication bias was found based on Egger's linear regression test. However, the Egger test revealed a possible publication bias for the association between duration of opium use and overall cancer risk. By using the application of the trim-and-fill method, the pooled ES for this association remained significant. Therefore, our results were not affected by publication bias. Sensitivity analysis showed that, after the exclusion of Nasrollahzadeh et al.'s study [21] from the analysis, the significant positive association between teriak use and overall cancer risk became nonsignificant (pooled ES: 2.16, 95% CI: 0.91–5.16, *P* = 0.08). In addition, excluding the study of Sheikh et al. [27] from the analysis of shireh use and overall cancer risk caused that the nonsignificant association became significant (pooled ES: 5.51, 95% CI: 2.75–11.06, *P* ≤ 0.01). For other associations, the pooled ESs did not depend on one study.

## 4. Discussion

In this study, we found the significant positive relationships between ever opium use and the risk of overall and individual types of cancers except for esophageal and colon cancers. In addition, the duration of consumption and opium doses were associated with an increased risk of overall cancer risk and the risk of different cancer types. However, the associations between opium doses and the risk of head and neck and larynx cancers were not significant. Also, we found that both opium ingestion and smoking were positively associated with overall cancer risk. In terms of routes of opium types, teriak use, but not shireh, was associated with an increased risk of overall and GI cancers.

The relationship between opium use and cancer risk has long been a research interest for researchers. Several observational studies have examined this association; however, findings from these studies are conflicting. In this meta-analysis, we found a positive association between opium use and the risk of overall and individual types of cancers. Such findings were also seen for opium doses and the duration of opium use. In line with our findings, a meta-analysis of opium use and bladder cancer risk in 2017 revealed a significant positive relationship [31]. Moreover, in a systematic review, Kamangar et al. concluded that opium use is an independent risk factor for esophagus, gastric, larynx, lung, and urinary bladder cancers [2]. Given the lack of significant association between ever opium use and esophagus cancer risk in this meta-analysis, our findings in this regard are in contrast to those reported from Kamangar et al.'s study. This inconsistency can be explained by the design and publication data of Kamangar's et al. study in which no meta-analysis was performed on the association between opium use and esophagus cancer risk. Also, since the publication of that review, two studies on the link between opium use and esophagus cancer risk were published [10, 11]. However, it must be kept in mind that the lack of significant association between opium use and esophagus cancer risk in the current meta-analysis may be due to the different quality of studies included in the association. Of three studies that assessed this association, only one with a low quality (quality score 5 of 9) and a low sample size reported no significant association [10], while two other studies with higher quality and a greater number of participants showed a significant positive association in this regard [11, 24]. Further studies are needed to reveal facts on the association.

In the current study, there was no difference between opium smoking and ingestion in relation to the risk of overall and individual types of cancers except for head and neck cancers. Opium ingestion, but not opium smoking, was associated with an increased risk of head and neck cancers. Although there were only two studies on the meta-analysis of opium routes and head and neck cancers [16, 27], the different findings on these routes might be due to the different substances produced through opium ingestion and smoking. Opium ingestion can expose consumers to a high amount of morphine and other alkaloids which all potentially affect the brain and nervous system [29]. However, smoking opium can also increase the levels of carcinogenic agents such as heterocyclic and polycyclic aromatic hydrocarbons and aromatic amines in different organs [47, 48].

Regarding opium types, we found that consuming teriak, but not shireh, was associated with a greater risk of overall and GI cancers. These differences might be explained by different processes used in teriak production compared with producing shireh [48]. Teriak is the air-dried extract of the opium poppy plant that is obtained from the ripened capsules of this plant. In contrast, shireh is produced with three additional processes compared to teriak which include boiling teriak of raw opium in water, filtering the mixture several times, and evaporating the filtrate [27, 48]. These additional processes may affect the compounds of opium and, therefore, alter the health effects of opium in the form of shireh.

Some mechanisms by which opium use increases the risk of cancer are suggested. It has been shown that opium pyrolysis during opium smoking produces multiple carcinogenic compounds including heterocyclic and polycyclic aromatic hydrocarbons, primary aromatic amines, and N-nitrosamines, which all can absorb through the respiratory system and induce their carcinogenic effects in different organs [29, 30, 49]. Also, some compounds produced through opium ingestion or smoking such as alkaloids may have genotoxic and mutagenic properties [29, 50]. Moreover, it has been proposed that opium plays a role in cancer promotion [51]. Some compounds produced during opium use can stimulate angiogenesis and neovascularization in tumors and also may activate cancer cell proliferation and migration [51]. Opium can also increase the effects of nonopium carcinogens through modifying the pharmacokinetics of these carcinogens, increasing their bioavailability, impairing the physiological function of target organs, and finally prolonging their exposure to the potential carcinogens [52].

This study is the first systematic review and meta-analysis that comprehensively assessed current evidence on the association between opium use and cancer risk. Also, different aspects of opium use including opium doses, duration of consumption, opium types, and routes of opium use were assessed in the current meta-analysis. Our findings also need to be interpreted by considering several limitations. First, since the studies included in the current meta-analysis were observational, mostly with a case-control design, causality cannot be established. Second, the effects of residual confounders including unmeasured behavioral and biological factors can affect the findings obtained in the current meta-analysis. Third, errors in the measurement of opium use and covariates cannot be entirely excluded owing to the observational design of included studies. Misclassification due to the measurement errors could result in an underestimation of the association between opium use and cancer risk. Fourth, there was evidence of considerable heterogeneity among included studies which might be explained by variations in the methods used for cancer ascertainment and variables adjusted in the statistical analysis. Finally, some included articles were of low-quality studies; however, we performed subgroup analysis based on the quality of studies to show the findings of high-quality studies separately.

In conclusion, we found that opium use was positively associated with the risk of overall and some individual types of cancers. Opium doses and duration of consumption were also involved in these associations such that higher doses and longer duration of opium use were associated with an increased risk of cancer. However, the associations between opium use and esophageal and colon cancers as well as the associations between opium doses and the risk of head and neck and larynx cancers were not significant. Since a limited number of studies were included in these associations, further studies are needed to confirm our findings for these types of cancers. Both routes of opium ingestion and smoking were associated with a greater risk of cancer. Regarding opium types, we found that using teriak, but not shireh, could increase the risk of cancer. Given the recent increase in using opium derivatives, further global proceedings to reduce the misuse and prevent hazardous long-term effects of opiates are urgently required.

## Figures and Tables

**Table 1:** Subgroup analyses for the association between opium use and cancer risk in adults aged ≥18 years.

	#ES^1^	Pooled ES (95% CI)^2^	*P* ^3^	*I*_2_ (%)^4^	*P*-heterogeneity^5^
*Ever versus never use of opium and cancer*					
Overall	20	3.53 (2.60–4.79)	≤0.01	89.5	≤0.01
Subgroup analysis					
Study design					
Cohort					
Case-control	2	1.86 (0.79–4.39)	0.15	75.3	0.04
Cancer type	18	3.71 (2.93–4.70)	≤0.01	73.8	≤0.01
GI	12	2.49 (1.81–3.43)	≤0.01	77.5	≤0.01
Bladder	6	3.85 (2.96–5.00)	≤0.01	26.9	0.23
Lung	3	5.00 (2.70–9.28)	≤0.01	66.2	0.05
Head and neck	3	4.35 (2.61–7.26)	≤0.01	60.3	0.08
Larynx	5	8.85 (6.16–12.74)	≤0.01	24.5	0.25
Oral	3	1.81 (1.23–2.65)	≤0.01	9.7	0.33
Pancreas	2	2.14 (1.37–3.36)	≤0.01	0	0.55
Esophageal	3	2.05 (0.93–4.55)	0.08	62.3	0.07
Colorectal	2	4.49 (2.81–7.16)	≤0.01	0	0.99
Colon	3	3.01 (0.89–10.11)	0.07	84.9	≤0.01
Gastric	4	2.38 (1.40–4.06)	≤0.01	74.0	≤0.01
Cancer assessment					
Medical records	6	4.27 (2.99–6.10)	≤0.01	33.8	0.18
Histological/pathological methods	14	3.24 (2.24–4.69)	≤0.01	92.0	≤0.01
Adjustment for tobacco use					
Yes	16	3.32 (2.35–4.69)	≤0.01	91.0	≤0.01
No	4	4.40 (2.63–7.36)	≤0.01	57.4	0.07
Adjustment for dietary factors					
Yes	9	4.35 (3.43–5.51)	≤0.01	0	0.62
No	11	2.97 (1.96–4.50)	≤0.01	93.6	≤0.01
Study quality					
High	15	3.18 (2.27–4.45)	≤0.01	90.5	≤0.01
Low	5	5.03 (2.43–10.43)	≤0.01	79.5	≤0.01
*Highest versus lowest doses of opium use and cancer*					
Overall	10	4.29 (2.15–8.54)	≤0.01	82.7	≤0.01
Subgroup analyses					
Cancer type					
GI	7	2.82 (1.31–6.05)	≤0.01	74.4	≤0.01
Lung	2	10.06 (3.84–26.35)	≤0.01	0	0.90
Head and neck	2	2.79 (0.28–27.88)	0.38	95.9	≤0.01
Larynx	2	3.33 (0.25–44.05)	0.36	95.5	≤0.01
Oral	2	0.86 (0.35–2.10)	0.73	0	0.33
Colorectal	2	7.40 (3.28–16.71)	≤0.01	0	0.81
Colon	2	8.34 (3.54–19.63)	≤0.01	0	0.77
Cancer assessment					
Medical records	5	8.15 (5.02–13.24)	≤0.01	0	0.40
Histological/pathological methods	5	2.47 (1.09–5.58)	0.03	81.1	≤0.01
Adjustment for tobacco use					
Yes	7	3.80 (1.44–10.02)	≤0.01	83.4	≤0.01
No	3	5.66 (1.92–16.63)	≤0.01	81.9	≤0.01
Adjustment for dietary factors					
Yes	6	7.94 (5.03–12.51)	≤0.01	0	0.53
No	4	2.04 (0.86–4.85)	0.10	82.6	≤0.01
Study quality					
High	9	3.87 (1.91–7.88)	≤0.01	80.9	≤0.01
Low	1	9.22 (4.19–20.28)	≤0.01	—	—
*Duration of opium use and cancer risk*					
Overall	11	3.74 (2.41–5.82)	≤0.01	75.8	≤0.01
Subgroup analysis					
Study design					
Cohort					
Case-control	1	1.81 (1.27–2.57)	≤0.01	—	—
Cancer type	10	4.23 (2.55–7.02)	≤0.01	74.8	≤0.01
GI	8	2.86 (1.69–4.83)	≤0.01	71.8	≤0.01
Lung	3	3.95 (2.37–6.60)	≤0.01	0	0.77
Head and neck	2	5.50 (1.09–27.72)	0.04	89.9	≤0.01
Larynx	2	5.77 (1.19–28.11)	0.03	87.4	≤0.01
Oral	2	2.03 (0.87–4.75)	0.10	0	0.91
Colorectal	2	6.99 (3.68–13.28)	≤0.01	0	0.61
Colon	2	8.24 (3.50–19.38)	≤0.01	0	0.80
Cancer assessment					
Medical records	5	9.26 (5.60–15.29)	≤0.01	0	0.81
Histological/pathological methods	6	2.25 (1.62–3.11)	≤0.01	52.3	0.06
Adjustment for tobacco use					
Yes	9	3.19 (2.05–4.97)	≤0.01	70.2	≤0.01
No	2	6.47 (1.74–24.03)	≤0.01	82.2	0.01
Adjustment for dietary factors					
Yes	6	8.34 (5.37–13.08)	≤0.01	0	0.81
No	5	2.02 (1.54–2.64)	≤0.01	31.1	0.21
Study quality					
High	10	3.18 (2.13–4.74)	≤0.01	68.1	≤0.01
Low	1	13.16 (5.32–32.54)	≤0.01	—	—
*Opium smoking versus never use*					
Overall	5	2.43 (1.46–4.04)	≤0.01	92.2	≤0.01
Cancer type					
GI	4	1.47 (1.07–2.02)	0.01	52.1	0.10
Bladder	2	3.55 (2.59–4.86)	≤0.01	0	0.36
Lung	2	3.00 (1.12–7.98)	0.02	70.7	0.06
Head and neck	2	1.45 (0.40–5.35)	0.57	88.4	≤0.01
Larynx	2	3.66 (2.24–5.97)	≤0.01	34.6	0.21
Oral	3	1.71 (0.88–3.32)	0.11	58.0	0.09
*Opium ingestion versus never use and cancer*					
Overall	5	2.66 (1.40–5.07)	≤0.01	92.5	≤0.01
Cancer type					
GI	4	1.82 (1.15–2.89)	0.01	47.4	0.12
Bladder	2	4.02 (2.96–5.47)	≤0.01	0	0.75
Lung	2	2.00 (1.05–3.83)	0.03	54.0	0.14
Head and neck	2	4.29 (1.11–16.62)	0.03	86.8	≤0.01
Larynx	2	6.82 (1.05–44.17)	0.04	89.6	≤0.01
Oral	3	2.59 (0.92–7.29)	0.07	20.7	0.28
*Teriak use versus never use and cancer*					
Overall	3	1.98 (1.08–3.62)	0.03	94.8	≤0.01
Cancer type					
GI	3	1.98 (1.08–3.62)	0.03	94.8	≤0.01
Head and neck	2	2.08 (0.73–5.89)	0.16	89.4	≤0.01
Larynx	2	3.97 (1.69–9.36)	≤0.01	76.4	0.04
*Shireh use versus never use and cancer*					
Overall	3	3.03 (0.82–11.14)	0.10	94.6	≤0.01
Cancer type					
GI	3	3.03 (0.82–11.14)	0.10	94.6	≤0.01
Larynx	2	7.54 (2.13–26.63)	≤0.01	69.7	0.07

GI: gastrointestinal, ES: effect size; ^1^number of effect sizes; ^2^obtained from the random-effects model; ^3^overall effect sizes, and ^4^inconsistency, the percentage of variation across studies due to heterogeneity; ^5^obtained from the Q-test.

## Data Availability

The datasets generated and/or analyzed during this study are available from the corresponding author upon reasonable request.

## Additional Points

What is already known about this topic?

Opium is an addictive substance that has been used for recreational or medical purposes. It has been estimated that 16.5 million individuals around the world are addicted to different types of opiates; of them, 4 million people use raw opium.

What does this article add?

Higher opium doses and longer duration of consumption were associated with an increased risk of overall and individual types of cancer. Among opium types, teriak, but not shireh, could increase the risk of cancer.

## Supplementary Materials

Supplementary Materials Supplemental Figure 1: flowchart of study selection. Supplemental Table 1: terms used to search articles on the association between opium use and cancer risk. Supplemental Table 2: characteristics of included studies on the association between opium use and cancer risk in adults aged >18 years. Supplemental Table 3: characteristics of included studies on the association between duration of opium use and cancer risk in adults aged >18 years. Supplemental Table 4: characteristics of included studies on the association between routes of opium use and cancer risk in adults aged >18 years. Supplemental Table 5: characteristics of included studies on the association between types of opium use and cancer risk in adults aged >18 years.
