# Supplementary material for: Opium Use and Cancer Risk: A Comprehensive Systematic Review and Meta-Analysis of Observational Studies
Source: Int J Clin Pract. 2022 Feb 18;2022:5397449. doi: 10.1155/2022/5397449 (PMC9159125; doi:10.1155/2022/5397449)
Supplement: Supplementary Materials — Supplemental Figure 1: flowchart of study selection. Supplemental Table 1: terms used to search articles on the association between opium use and cancer risk. Supplemental Table 2: characteristics of included studies on the association between opium use and cancer risk in adults aged >18 years. Supplemental Table 3: characteristics of included studies on the association between duration of opium use and cancer risk in adults aged >18 years. Supplemental Table 4: characteristics of included studies on the association between routes of opium use and cancer risk in adults aged >18 years. Supplemental Table 5: characteristics of included studies on the association between types of opium use and cancer risk in adults aged >18 years. [file 5397449.f1.docx]

**Supplemental Figure 1:** Flowchart of study selection

**
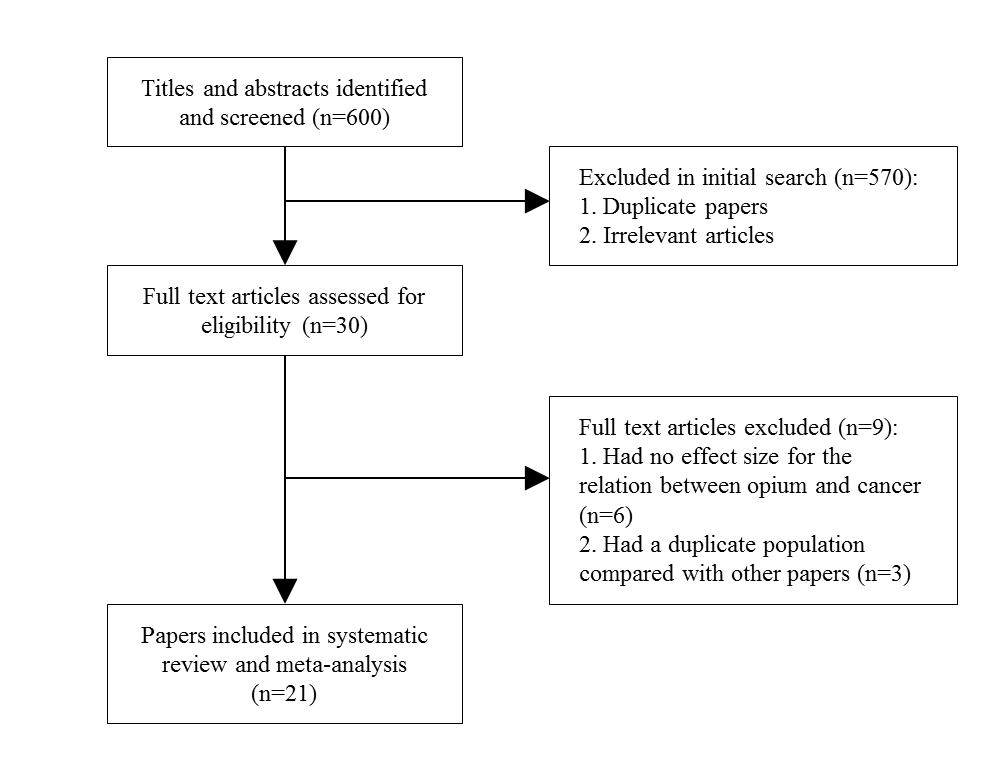
**

**Supplemental Table 1:** Terms used to search articles on the association between opium use and cancer risk

| **Concept 1** | (Opium OR Papaveretum OR Omnopon OR Pantopon OR Papaver OR Papaver OR Poppy OR Poppies) |
| --- | --- |
| **Concept 2** | (Neoplasms OR Neoplasms OR Neoplasia OR Cancer OR Malignancy OR Tumor OR Carcinoma OR Carcinoma) |
| **Search** | Concept 1 AND Concept 2 |

**Supplemental Table 2**: Characteristics of included studies on the association between opium use and cancer risk in adults aged >18 years

| Author | Design | Age* | Sample size | Cases | Exposure^**^ | Outcome | Outcome assessment | Comparisons | ES (95%CI) | Adjustments |
| --- | --- | --- | --- | --- | --- | --- | --- | --- | --- | --- |
| Sadjadi et al. 2014 | Cohort | >40 | M/F: 928 | 36 | Opium: unclear | Gastric cancer | Histological methods | Ever vs. never | HR: 3.24 (1.37-7.66) | Age, gender, family history of cancer, smoking, alcohol, fruit, vegetable, and salt intake |
| Sheikh et al. 2020 | Cohort | 40-75 | M/F: 50045 | 1833  914  342  308  116  95  80  78  73  47  38  86  44  42  42  32  31  25  23 | Opium: teriak, shireh, sukhteh, heroin | all cancers  GI cancers  Esophageal cancer  Gastric cancer  Lung cancer  Colon cancer  Brain cancer  Pancreatic cancer  Liver cancer  Bladder cancer  Laryngeal cancer  Breast cancer  Leukemia  Lymphoma  Ovarian cancer  Prostate cancer  Oropharyngeal cancers  Cervical cancer  Rectal cancer | Histological methods | Opium users but not tobacco vs. neither opium nor tobacco users | HR: 1.32 (1.13-1.54)  HR: 1.27 (1.03-1.57)  HR: 1.41 (1.02-1.96)  HR: 1.33 (0.96-1.86)  HR: 7.34 (4.43-12.13)  HR: 0.89 (0.40-2.00)  HR: 2.83 (1.27-6.29)  HR: 2.52 (1.25-5.07)  HR: 2.05 (1.03-4.07)  HR: 4.21 (1.87-9.46)  HR: 17.75 (6.06-51.94)  HR: 1.55 (0.70-3.43)  HR: 1.61 (0.78-3.33)  HR: 1.47 (0.67-3.20)  HR: 0.77 (0.18-3.27)  HR: 0.62 (0.24-1.57)  HR: 1.58 (0.65-3.81)  HR: 1.46 (0.42-5.00)  HR: 0.94 (0.32-2.74) | Age, sex, ethnicity, smoking, alcohol, residence |
| Tahami et al. 2014 | Case-control | Cases: 61.9, controls: 63.2 | M/F: 426 | 142  89 | Opium: teriak, shireh, sukhteh, heroin | Upper GI cancers  gastric cancer | Pathological methods | Ever vs. never | OR: 14.00 (4.70-47.50)  OR: 13.00 (4.20-41.90) | Age, gender, place, intakes of meat, fruits, vegetables, hydrogenated fats, and smoking |
|  |  |  |  |  |  | Upper GI cancers  gastric cancer |  | >median vs. never | OR: 12.00 (3.20-44.10)  OR: 10.50 (2.40-46.10) |  |
| Akbari et al. 2015 | Case-control | Cases: 66.2, controls: 65.9 | M/F: 585 | 198 | Opium: teriak, shireh, sukhteh, heroin | Bladder cancer | Medical records | Ever vs. never | OR: 2.40 (0.60-9.40) | Age, gender, intakes of red meat, poultry, fish, hydrogenated fat, olive oil, butter, fat, fruits, nuts, moldy food, alcohol, tobacco use |
|  |  |  |  |  |  | Bladder cancer |  | >median vs. never | OR: 6.00 (1.10-34.70) |  |
| Mohebbi et al. 2020 | Case-control | >20 | M/F: 3728 | 663  254  54  327 | Opium: teriak, shireh, sukhteh | Head and neck cancers  Lip & oral cancer  Pharynx cancer  Larynx cancer | Pathological methods | Ever vs. never | OR: 3.76 (2.96-4.79)  OR: 1.53 (0.97-2.41)  OR: 2.90 (1.40-6.02)  OR: 6.55 (4.69-9.13) | Age, gender, place, cigarette smoking, water-pipe smoking, regular alcohol use, socioeconomic status, oral health |
|  |  |  |  |  |  | Head and neck cancers  Lip & oral cancer  Pharynx cancer  Larynx cancer |  | T3 vs. T1 | OR: 0.88 (0.53-1.44)  OR: 1.12 (0.39-3.19)  OR: 0.41 (0.07-2.26)  OR: 0.92 (0.53-1.60) |  |
| Tahami et al. 2020 | Case-control | >40 | M/F: 420 | 140 | Opium: teriak, shireh, sukhteh, heroin | Lung cancer | Medical records | Ever vs. never | OR: 5.95 (1.87-18.92) | Age, gender, intakes of meat, fruit and vegetables, hydrogenated fats, olive oil, smoking, alcohol, education |
|  |  |  |  |  |  | Lung cancer |  | >median vs. never | OR: 9.36 (2.05-42.72) |  |
| Nasrollahzadeh et al. 2008 | Case-control | Cases: 65.4, controls: 64.3 | M/F: 871 | 300 | Opium: teriak, shireh, sukhteh, heroin | Esophageal cancer | Histological methods | >median vs. never | OR: 2.38 (1.47-3.85) | Age, gender, place, education, ethnicity |
| Shakhssalim et al. 2010 | Case-control | Cases: 65.64, controls: 64.67 | M/F: 1384 | 692 | Opium: unclear | bladder cancer | Medical records | Ever vs. never | OR: 2.57 (1.55-4.26) | Age, sex, smoking |
| Alizadeh et al. 2020 | Case-control | >40 | M/F: 420 | 140 | Opium: teriak, shireh, sukhteh, heroin | Head and neck cancers  Laryngeal cancer | Medical records | Ever vs. never | OR: 8.13 (4.08-16.21)  OR: 11.98 (5.05-28.39) | Age, gender, intakes of meat, fruit and vegetables, hydrogenated fats, olive oil, education |
|  |  |  |  |  |  | Head and neck cancers  Laryngeal cancer |  | >median vs. never | OR: 9.22 (4.19-20.28)  OR: 12.82 (4.96-33.11) |  |
| Hosseini et al. 2010 | Case-control | Cases: 62.05, controls: 62.0 | M/F: 358 | 179 | Opium: unclear | Bladder cancer | Pathological methods | Ever vs. never | OR: 4.57 (3.82-5.47) | - |
| Bakhshaee et al. 2017 | Case-control | >40 | M/F: 181 | 58  95 | Opium: unclear | Laryngeal cancer  Esophageal cancer | Biopsy | Ever vs. never | OR: 6.06 (1.1-33.23)  OR: 1.44 (0.57-3.62) | Age, smoking |
| Masjedi et al. 2013 | Case-control | Cases: 59.9, controls: 59.4 | M/F: 726 | 242 | Opium: unclear | Lung cancer | Histological methods | Ever vs. never | OR: 3.23 (2.07-5.03) | Age, gender, place, education, ethnicity |
|  |  |  |  |  |  | Lung cancer |  | >median vs. never | OR: 10.56 (3.04-36.66) |  |
| Mousavi et al. 2003 | Case-control | Cases: 57.5, controls: 56.7 | M/F: 410 | 98 | Opium: unclear | Laryngeal Cancer | Histological methods | Ever vs. never | OR: 10.74 (5.76-20.02) | Age, gender, smoking |
| Shakeri et al. 2012 | Case-control | >35 | M/F: 1261 | 430 | Opium: teriak, shireh, sukhteh, heroin | Esophageal cancer | Histological methods | Ever vs. never | OR: 1.48 (1.06-2.06) | Age, gender, cigarette smoking, nass and hookah use, ethnicity, education, place |
| Shakeri et al. 2013 | Case-control | Cases: 65.2, controls: 63.6 | M/F: 922 | 309 | Opium: teriak, shireh, sukhteh, heroin | Gastric cancer | Biopsy | Ever vs. never | OR: 3.10 (1.90-5.20) | Age, gender, place, ethnicity, education, intakes of fruit, vegetable, socioeconomic status, cigarette, hookah and nass use |
| Shakeri et al. 2016 | Case-control | Cases: 64.6, controls: 64.7 | M/F: 685 | 357 | Opium: teriak, shireh, sukhteh, heroin | Pancreatic cancer | Endoscopy | Ever vs. never | OR: 1.91 (1.06-3.43) | Age, sex, residence, alcohol use, tobacco use |
|  |  |  |  |  |  | Pancreatic cancer |  | >median vs. never | OR: 1.19 (0.41-3.51) |  |
| Ghadimi et al. 2015 | Case-control | Cases: 63.6, controls: 61.8 | M/F: 304 | 152 | Opium: unclear | Bladder cancer | Histological methods | Ever vs. never | OR: 8.82 (1.99-39.08) | Age, sex, place |
| Lotfi et al. 2016 | Case-control | Cases: 61.5, controls: 61.4 | M/F: 400 | 200 | Opium: unclear | Bladder cancer | Histological methods | Ever vs. never | OR: 3.01 (1.73-5.23) | Age, sex, place |
| Vazirnejad et al. 2020 | Case-control | Cases: 61.5, controls: 61.4 | M/F: 280 | 90 | Opium: unclear | GI cancer | Pathological methods | Ever vs. never | OR: 5.94 (2.37-14.99) | Age, gender, place, smoking, education, family history of cancer, intakes of red meat, fruit, vegetables |
| lankarani et al. 2017 | Case-control | >40 | M/F: 480 | 160  93 | Opium: teriak, shireh, sukhteh, heroin | Colorectal cancer  Colon cancer | Pathological methods | Ever vs. never | OR: 4.48 (2.27-8.82)  OR: 5.4 (2.19-13.55) | Age, gender, place, intakes of meat, fruit, vegetables, hydrogenated fats, smoking |
|  |  |  |  |  |  | Colorectal cancer  Colon cancer |  | >median vs. never | OR: 6.53 (1.75-24.41)  OR: 7.11 (1.78-28.39) |  |
| Tahami et al. 2016 | Case-control | >40 | M/F: 525 | 350 | Opium: teriak, shireh, sukhteh, heroin | Colorectal cancer  Colon cancer | Medical records | Ever vs. never | OR: 4.50 (2.40-8.70)  OR: 5.70 (2.70-11.9) | Age, gender, place, intakes of meat, fruit, vegetables, hydrogenated fats, smoking |
|  |  |  |  |  |  | Colorectal cancer  Colon cancer |  | >median vs. never | OR: 8.00 (2.80-22.20)  OR: 9.20 (3.10-27.40) |  |

Abbreviations: GI: gastrointestinal, T: tertile, M: male, F: female, ES: effect size, HR: hazard ratio, RR: risk ratio, OR: odds ratio

^*^Presented as mean or range

^**^teriak: raw opium, shireh: opium sap, sukhteh: burned opium

**Supplemental Table 3**: Characteristics of included studies on the association between duration of opium use and cancer risk in adults aged >18 years

| Author | Design | Age* | Sample size | Cases | Exposure^**^ | Outcome | Outcome assessment | Comparisons | ES (95%CI) | Adjustments |
| --- | --- | --- | --- | --- | --- | --- | --- | --- | --- | --- |
| Sheikh et al. 2020 | Cohort | 40-75 | M/F: 50045 | 1833  914  116 | Opium: teriak, shireh, sukhteh, heroin | all cancers  GI cancers  Lung cancer | Histological methods | >30 y vs. never | HR: 1.81 (1.27-2.56)  HR: 1.56 (0.99-2.48)  HR: 4.83 (1.65-14.01) | Age, sex, ethnicity, smoking, alcohol, residence |
| Tahami et al. 2014 | Case-control | Cases: 61.9, controls: 63.2 | M/F: 426 | 142  89 | Opium: teriak, shireh, sukhteh, heroin | Upper GI cancers  gastric cancer | Pathological methods | >median vs. never | OR: 12.00 (3.20-44.10)  OR: 10.50 (2.40-46.10) | Age, gender, place, intakes of meat, fruits, vegetables, hydrogenated fats, and smoking |
| Akbari et al. 2015 | Case-control | Cases: 66.2, controls: 65.9 | M/F: 585 | 198 | Opium: teriak, shireh, sukhteh, heroin | Bladder cancer | Medical records | >median vs. never | OR: 6.00 (1.10-34.70) | Age, gender, intakes of red meat, poultry, fish, hydrogenated fat, olive oil, butter, fat, fruits, nuts, moldy food, alcohol, tobacco use |
| Mohebbi et al. 2020 | Case-control | >20 | M/F: 3728 | 663  254  54  327 | Opium: teriak, shireh, sukhteh | Head and neck cancers  Lip & oral cancer  Pharynx cancer  Larynx cancer | Pathological methods | T3 vs. T1 | OR: 2.52 (1.55-4.11)  OR: 2.09 (0.75-5.80)  OR: 1.90 (0.40-8.60)  OR: 2.71 (1.56-4.68) | Age, gender, place, cigarette smoking, water-pipe smoking, regular alcohol use, socioeconomic status, oral health |
| Tahami et al. 2020 | Case-control | >40 | M/F: 420 | 140 | Opium: teriak, shireh, sukhteh, heroin | Lung cancer | Medical records | >median vs. never | OR: 5.50 (1.32-22.91) | Age, gender, intakes of meat, fruit and vegetables, hydrogenated fats, olive oil, smoking, alcohol, education |
| Alizadeh et al. 2020 | Case-control | >40 | M/F: 420 | 140 | Opium: teriak, shireh, sukhteh, heroin | Head and neck cancers  Laryngeal cancer | Medical records | >median vs. never | OR: 13.16 (5.32-32.53)  OR: 13.68 (5.12-36.56) | Age, gender, intakes of meat, fruit and vegetables, hydrogenated fats, olive oil, education |
| Masjedi et al. 2013 | Case-control | Cases: 59.9, controls: 59.4 | M/F: 726 | 242 | Opium: unclear | Lung cancer | Histological methods | >median vs. never | OR: 3.44 (1.81-6.52) | Age, gender, place, education, ethnicity |
| Shakeri et al. 2012 | Case-control | >35 | M/F: 1261 | 430 | Opium: teriak, shireh, sukhteh, heroin | Esophageal cancer | Histological methods | >median vs. never | OR: 1.50 (0.99-2.28) | Age, gender, cigarette smoking, nass and hookah use, ethnicity, education, place |
| Shakeri et al. 2016 | Case-control | Cases: 64.6, controls: 64.7 | M/F: 685 | 357 | Opium: teriak, shireh, sukhteh, heroin | Pancreatic cancer | Endoscopy | >median vs. never | OR: 1.79 (0.81-3.97) | Age, sex, residence, alcohol use, tobacco use |
| lankarani et al. 2017 | Case-control | >40 | M/F: 480 | 160  93 | Opium: teriak, shireh, sukhteh, heroin | Colorectal cancer  Colon cancer | Pathological methods | >median vs. never | OR: 5.83 (2.23-15.21)  OR: 7.20 (1.87-28.26) | Age, gender, place, intakes of meat, fruit, vegetables, hydrogenated fats, smoking |
| Tahami et al. 2016 | Case-control | >40 | M/F: 525 | 350 | Opium: teriak, shireh, sukhteh, heroin | Colorectal cancer  Colon cancer | Medical records | >median vs. never | OR: 8.10 (2.60-14.60)  OR: 9.00 (3.00-27.20) | Age, gender, place, intakes of meat, fruit, vegetables, hydrogenated fats, smoking |

Abbreviations: GI: gastrointestinal, T: tertile, M: male, F: female, ES: effect size, HR: hazard ratio, RR: risk ratio, OR: odds ratio

^*^Presented as mean or range

^**^teriak: raw opium, shireh: opium sap, sukhteh: burned opium

**Supplemental Table 4**: Characteristics of included studies on the association between routes of opium use and cancer risk in adults aged >18 years

| Author | Design | Age* | Sample size | Cases | Exposure^**^ | Outcome | Outcome assessment | Comparisons | ES (95%CI) | Adjustments |
| --- | --- | --- | --- | --- | --- | --- | --- | --- | --- | --- |
| Sheikh et al. 2020 | Cohort | 40-75 | M/F: 50045 | 1833  914  342  308  116  95  80  78  73  47  38  86  44  42  42  32  31  25  23 | Opium: teriak, shireh, sukhteh, heroin | all cancers  GI cancers  Esophageal cancer  Gastric cancer  Lung cancer  Colon cancer  Brain cancer  Pancreatic cancer  Liver cancer  Bladder cancer  Laryngeal cancer  Breast cancer  Leukemia  Lymphoma  Ovarian cancer  Prostate cancer  Oropharyngeal cancers  Cervical cancer  Rectal cancer | Histological methods | Opium smoking vs. never use | HR: 1.31 (1.14-1.50)  HR: 1.27 (1.04-1.55)  HR: 1.37 (1.00-1.88)  HR: 1.43 (1.04-1.96)  HR: 1.96 (1.18-3.26)  HR: 0.82 (0.39-1.72)  HR: 0.70 (0.30-1.64)  HR: 1.67 (0.86-3.23)  HR: 1.00 (0.44-2.28)  HR: 2.61 (1.25-5.44)  HR: 2.46 (1.11-5.50)  HR: 1.53 (0.61-3.85)  HR: 1.56 (0.68-3.60)  HR: 1.48 (0.62-3.56)  HR: 0.59 (0.08-4.35)  HR: 0.54 (0.16-1.85)  HR: 1.69 (0.58-4.49)  HR: 5.11 (1.47-17.72)  HR: 0.31 (0.04-2.39) | Age, sex, ethnicity, smoking, alcohol, residence |
|  |  |  |  | 1833  914  342  308  116  95  80  78  73  47  38  86  44  42  32  31  23 |  | all cancers  GI cancers  Esophageal cancer  Gastric cancer  Lung cancer  Colon cancer  Brain cancer  Pancreatic cancer  Liver cancer  Bladder cancer  Laryngeal cancer  Breast cancer  Leukemia  Lymphoma  Prostate cancer  Oropharyngeal cancers  Rectal cancer |  | Opium ingestion vs. never use | HR: 1.49 (1.25-1.78)  HR: 1.33 (1.04-1.71)  HR: 1.18 (0.78-1.79)  HR: 1.34 (0.88-2.05)  HR: 2.72 (1.54-4.80)  HR: 1.52 (0.59-3.92)  HR: 2.09 (0.94-4.62)  HR: 1.41 (0.57-3.48)  HR: 2.85 (1.37-5.92)  HR: 3.55 (1.51-8.35)  HR: 2.55 (0.96-6.75)  HR: 1.85 (0.44-7.70)  HR: 1.73 (0.57-5.18)  HR: 1.73 (0.50-5.98)  HR: 0.88 (0.25-3.04)  HR: 0.65 (0.08-5.05)  HR: 1.46 (0.31-6.76) |  |
| Mohebbi et al. 2020 | Case-control | >20 | M/F: 3728 | 663  254  54  327 | Opium: teriak, shireh, sukhteh | Head and neck cancers  Lip & oral cancer  Pharynx cancer  Larynx cancer | Pathological methods | Opium smoking vs. never use | OR: 2.66 (2.03-3.47)  OR: 1.09 (0.64-1.86)  OR: 3.04 (1.43-6.47)  OR: 4.28 (2.98-6.14) | Age, gender, place, cigarette smoking, water-pipe smoking, regular alcohol use, socioeconomic status, oral health |
|  |  |  |  |  |  | Head and neck cancers  Lip & oral cancer  Pharynx cancer  Larynx cancer |  | Opium ingestion vs. never use | OR: 8.33 (4.67-14.85)  OR: 4.25 (1.45-11.69)  OR: 2.67 (0.33-21.57)  OR: 17.17 (8.44-34.91) |  |
| Nasrollahzadeh et al. 2008 | Case-control | Cases: 65.4, controls: 64.3 | M/F: 871 | 300 | Opium: teriak, shireh, sukhteh, heroin | Esophageal cancer | Histological methods | Opium smoking vs. never use | OR: 1.67 (1.06-2.63) | Age, gender, place, education, ethnicity |
|  |  |  |  |  |  | Esophageal cancer |  | Opium ingestion vs. never use | OR: 1.90 (1.09-3.32) |  |
| Hosseini et al. 2010 | Case-control | Cases: 62.05, controls: 62.0 | M/F: 358 | 179 | Opium: unclear | Bladder cancer | Pathological methods | Opium smoking vs. never use | OR: 3.80 (2.74-5.48) | - |
|  |  |  |  |  |  | Bladder cancer |  | Opium ingestion vs. never use | OR: 4.10 (3.22-6.22) |  |
| Masjedi et al. 2013 | Case-control | Cases: 59.9, controls: 59.4 | M/F: 726 | 242 | Opium: unclear | Lung cancer | Histological methods | Opium smoking vs. never use | OR: 5.40 (2.10-14.00) | Age, gender, place, education, ethnicity |
|  |  |  |  |  |  | Lung cancer |  | Opium ingestion vs. never use | OR: 1.40 (0.70-2.70) |  |

Abbreviations: GI: gastrointestinal, T: tertile, M: male, F: female, ES: effect size, HR: hazard ratio, RR: risk ratio, OR: odds ratio

^*^Presented as mean or range

^**^teriak: raw opium, shireh: opium sap, sukhteh: burned opium

**Supplemental Table 5**: Characteristics of included studies on the association between types of opium use and cancer risk in adults aged >18 years

| Author | Design | Age* | Sample size | Cases | Exposure^**^ | Outcome | Outcome assessment | Comparisons | ES (95%CI) | Adjustments |
| --- | --- | --- | --- | --- | --- | --- | --- | --- | --- | --- |
| Sheikh et al. 2020 | Cohort | 40-75 | M/F: 50045 | 1833  914  342  308  116  95  80  78  73  47  38 | Opium: teriak, shireh, sukhteh, heroin | all cancers  GI cancers  Esophageal cancer  Gastric cancer  Lung cancer  Colon cancer  Brain cancer  Pancreatic cancer  Liver cancer  Bladder cancer  Laryngeal cancer | Histological methods | Teriak use vs. never use | HR: 1.40 (1.23-1.58)  HR: 1.37 (1.15-1.62)  HR: 1.43 (1.09-1.89)  HR: 1.42 (1.07-1.88)  HR: 2.19 (1.41-3.40)  HR: 0.97 (0.52-1.82)  HR: 1.17 (0.62-2.20)  HR: 1.48 (0.81-2.68)  HR: 1.38 (0.77-2.46)  HR: 2.51 (1.25-5.04)  HR: 2.38 (1.10-5.12) | Age, sex, ethnicity, smoking, alcohol, residence |
|  |  |  |  | 1833  914  342  308  116  95  78  47  38 |  | all cancers  GI cancers  Esophageal cancer  Gastric cancer  Lung cancer  Colon cancer  Pancreatic cancer  Bladder cancer  Laryngeal cancer |  | Shireh use vs. never use | HR: 1.18 (0.84-1.66)  HR: 0.96 (0.57-1.62)  HR: 0.92 (0.37-2.26)  HR: 1.26 (0.58-2.72)  HR: 1.25 (0.38-4.12)  HR: 0.64 (0.08-4.71)  HR: 1.58 (0.37-6.73)  HR: 5.80 (1.86-18.03)  HR: 3.40 (0.92-12.55) |  |
| Mohebbi et al. 2020 | Case-control | >20 | M/F: 3728 | 663  254  54  327 | Opium: teriak, shireh, sukhteh | Head and neck cancers  Lip & oral cancer  Pharynx cancer  Larynx cancer | Pathological methods | Teriak use vs. never use | OR: 3.4 (2.64-4.37)  OR: 1.41 (0.87-2.27)  OR: 2.81 (1.32-5.97)  OR: 5.77 (4.09-8.15) | Age, gender, place, cigarette smoking, water-pipe smoking, regular alcohol use, socioeconomic status, oral health |
|  |  |  |  |  |  | Head and neck cancers  Lip & oral cancer  Pharynx cancer  Larynx cancer |  | Shireh use vs. never use | OR: 7.17 (4.44-11.58)  OR: 2.90 (1.05-7.97)  OR: 3.77 (0.80-17.68)  OR: 12.69 (7.25-22.22) |  |
| Nasrollahzadeh et al. 2008 | Case-control | Cases: 65.4, controls: 64.3 | M/F: 871 | 300 | Opium: teriak, shireh, sukhteh, heroin | Esophageal cancer | Histological methods | Teriak use vs. never use | OR: 1.62 (1.09-2.40) | Age, gender, place, education, ethnicity |
|  |  |  |  |  |  | Esophageal cancer |  | Shireh use vs. never use | OR: 3.41 (1.35-8.60) |  |

Abbreviations: GI: gastrointestinal, T: tertile, M: male, F: female, ES: effect size, HR: hazard ratio, RR: risk ratio, OR: odds ratio

^*^Presented as mean or range

^**^teriak: raw opium, shireh: opium sap, sukhteh: burned opium
